# Does Intra-Wound Vancomycin Powder Affect the Action of Intra-Articular Tranexamic Acid in Total Joint Replacement?

**DOI:** 10.3390/microorganisms8050671

**Published:** 2020-05-06

**Authors:** Antonios A. Koutalos, Athanasios Drakos, Apostolos Fyllos, Nikos Doxariotis, Sokratis Varitimidis, Konstantinos N. Malizos

**Affiliations:** Department of Orthopaedic Surgery and Musculoskeletal Trauma, University of Thessaly, 41500 Larissa, Greece; akoutmed@gmail.com (A.A.K.); drakosth@hotmail.com (A.D.); apofyl@hotmail.com (A.F.); nik-dox@otenet.gr (N.D.); svaritimidis@hotmail.com (S.V.)

**Keywords:** tranexamic acid, vancomycin, total hip arthroplasty, total knee arthroplasty, topical

## Abstract

The intra-articular use of tranexamic acid (TXA) has contributed to reduced blood loss in total joint arthroplasty (TJA). The purpose of this study is to assess the efficacy of simultaneously topical use of tranexamic acid and vancomycin powder within the TJA space. From 2016 to 2017, 219 consecutive total hip arthroplasties (THAs) and 272 total knee arthroplasties (TKAs) were performed in a tertiary care center, with a group of patients receiving intra-articular TXA and vancomycin, compared to patients receiving only TXA and to a control group that did not receive anything. Haemoglobin and hematocrit were measured preoperatively, on the first and third days. Transfusions and adverse events were recorded. Haemoglobin and hematocrit dropped significantly in all THA and TKA groups till the third day postoperatively, with a major reduction in the control group, compared to the other two treatment groups. Infections and thromboembolic events were similar in either group of the TJAs. In conclusion, the topical use of tranexamic acid mixed with the vancomycin powder within the joint space after the TJAs of the hip and knee did not alter the anti-fibrinolytic effect of TXA.

## 1. Introduction

The use of tranexamic acid (TXA) systemically and /or locally in total joint arthroplasties (TJAs) has been a widely established approach in clinical practice. The safety and the efficacy in reducing blood loss and the need for transfusions have been demonstrated in several randomised trials and meta-analyses [1,2,3,4,5]. TXA can be delivered either intravenously or topically within the joint space prior to the wound closure. A number of studies have also been used, both intravenous (IV) and intra-articularly combined. Both ways of administration are effective, but the local application of TXA has the advantage of providing higher doses while minimising any systemic adverse reactions such as seizures or thromboembolic events in high-risk patients [6,7,8]. Furthermore, local TXA is thought to have anti-inflammatory effects, thus positively affecting the patient recovery [9].

Vancomycin is an antibiotic that works primarily by inhibiting the formation of the bacterial cell wall. It also has an effect on the permeability of the bacterial cell membrane and RNA synthesis. Vancomycin is administered orally, intravenously, intra-peritoneally and, based on limited data, via the intraventricular, intrathecal and intravitreal routes. In an experimental study in rats tibiae, vancomycin was able to inhibit the development of osteomyelitis if the treatment was administered locally at the same time as a bacterial inoculum of 6.62 × 10^7^ CFU/mL, when compared to an untreated group. These findings suggest that local intramedullary vancomycin delivery could achieve sufficiently high local concentrations to prevent the development of osteomyelitis while minimising systemic toxicity [10]. Vancomycin powder has also been used in bone cement mix to achieve high concentration locally for the management of periprosthetic joint infections [11,12]. On the other hand, the local use of vancomycin powder in spine surgery has resulted in a decrease of the superficial and deep infections [13,14,15]. In our institution, where a high prevalence of community acquired-MRSA infections were observed [16], in a number of total hip and knee replacements in addition to the preoperative IV antibiotic prophylaxis, we have supplemented it by local administration of 2 g of vancomycin powder within the joint space prior to wound closure.

The purpose of this study is to assess the efficacy of the intra-articular combined administration of vancomycin powder and TXA inside the joint space, before the wound closure. Our hypothesis is that adding the vancomycin powder into the joint space together with TXA will not affect its anti-fibrinolytic effect.

## 2. Materials and Methods

From January 2016 to February 2017, we conducted a prospective non-randomised study with 219 consecutive patients who underwent primary THAs and consecutive 272 patients with primary TKAs. Patients with a history of allergy to vancomycin, undergoing revision surgery, with chronic kidney disease or liver dysfunction, with haemophilia or with a recent history of thromboembolism, recent acute myocardial infraction or stroke, or thrombophilia were excluded. According to the treating surgeon’s preference, the patients were allocated in three groups. Group A: those with intra-articular TXA prior to the wound closure, with 63 THAs and 87 TKAs; Group B: with intra-articular TXA combined with 2 g of vancomycin powder, with 67 THAs and 86 TKAs; Group C comprised of 89 THAs and 99 TKAs, without TXA or vancomycin powder into the joint space. The endpoints of the study were (1) the haemoglobin (Hgb), which was the primary endpoint and hematocrit (Hct) measured preoperatively, the 1st and the 3rd day postoperatively; (2) the number of transfusions in each group; (3) the surgical site infections (SSIs); (4) the thromboembolic episodes till the end of the second year after the operations. The findings of patients assigned to Groups A and B were compared with those of Group C. TXA has been regularly used since 2011 in more than one-third of the TJAs, and into long bone fracture fixations in our centre without adverse events. Our institutional scientific and ethics committee approved the protocol as a safety study (26.11.13/52967). Patients were informed preoperatively about the benefits and risks of topical administration of the study medications, both of which had been used topically in previous studies but not in combination and all gave their consent. Routine preoperative full blood count included Hgb, Hct, glucose, liver and kidney function serum markers, which were repeated on the first and the third postoperative days. All patients were operated under spinal anaesthesia by adult reconstruction fellowship-trained surgeons. All THAs were performed through a posterior approach and the TKAs through a classic medial para-patellar approach with tourniquet use. After insertion of all the implants and before wound closure, three grams of TXA [17] and two grams of vancomycin powder were placed into the joint space. Routine follow-up was scheduled and patients were evaluated at 6 weeks, 3 months, 1 year and 2 years. At each follow-up visit, patients were examined for signs of superficial or deep infection, including discomfort, warmth, redness, swelling, increasing pain, fever, or presence of sinus. The minimum follow-up was two years.

### 2.1. Per-Operative Care and Rehabilitation

Patients were mobilised on crutches the next day, bearing as much weight as tolerated. Haematocrit and haemoglobin were measured on the 1st and 3rd postoperative days during routine blood counts. Patients were transfused with red blood cells if haemoglobin dropped below 8 gr/dL or below 9 gr/dL in symptomatic patients, according to the department’s strict protocol [18].

Superficial SSI was diagnosed according to WHO criteria [19] and deep infection according to the Musculoskeletal Infection Society (MSIS) criteria [20]. The investigation of possible deep infection was done according to the AAOS algorithm [21]. Adverse events, including infection (superficial or deep) or deep vein thrombosis, were recorded. Patients but not the surgeons were blind to the TXA or vancomycin status. The primary outcome was the drop in Hgb on the third postoperative day between groups. Secondary outcomes were the drop in Hgb on the first day, as well as the changes in Hct and the volume of transfusions. Adverse events were compared between groups.

### 2.2. Statistical Analysis and Sample Size

Statistical analysis was performed with SPSS 20.Descriptive statistics were presented with mean and standard deviation. ANOVA was used to investigate if there was any difference between groups and specific differences were examined with the Tukey HSD test. Transfusion and adverse events were compared with Fisher’s exact test.

Considering that a difference of 0.5 g/dL (and a standard deviation on 1) in reductions of Hgb between groups would be clinically significant, this study with 60 or more patients in each group had more than 80% power to detect that difference [22].

## 3. Results

Patients with THAs were discharged after a mean of 4.3 (±1.2) days and patients with TKAs after 4.7 (±1.3) days. Twenty-three patients were lost to follow-up. There were ten patients in the THA group, including 4 in Group A, 2 in Group B, and 4 in Group C. In the TKA group, 13 patients, including 4 in Group A, 3 in Group B, and 6 in Group C, were also lost to follow up. Preoperatively there was no difference between the three groups as far as sex, age, Hct and Hgb are concerned. Demographics are presented in Table 1.

### 3.1. Total Hip Arthroplasty Results

**Figure 1:**
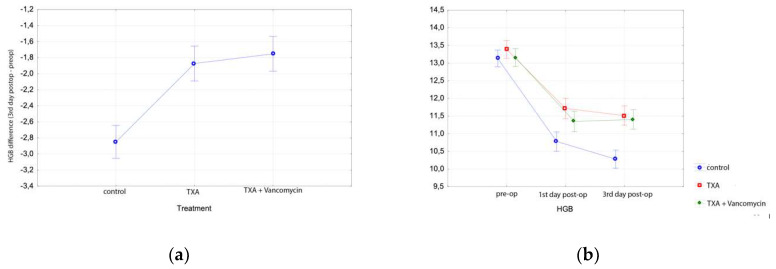
Results in total hip arthroplasty patients: (**a**) Hemoglobin difference between preoperative and 3rd day postoperative value in the control and treatment groups of total hip arthroplasty; (**b**) Hemoglobin levels before total hip arthroplasty, at the first and third postoperative days in the control and treatment groups.

Hgb dropped significantly on the 3rd postoperative day, as expected in all groups (Tukey HSD test, *p* < 0.001 for all groups), with a bigger drop in Group C than in Groups A and B (Table 2, Figure 1). Specifically, Hgb decreased by 3.3 ± 1.8 gr/dL in Group C, 2.5 ± 0.7 in Group A and 2.4 ± 1 in Group B, respectively (Tukey HSD, *p* < 0.001 for both comparisons). There was no difference in Hgb drop at the 3rd postoperative day between Groups A and B (Tukey HSD test, *p* = 0.811), supporting our hypothesis that the addition of vancomycin does not affect the topical action of TXA. Table 2 presents the mean Hgb values in all three groups preoperatively, on days 1 and 3 postoperatively (Figure 1).

Postoperatively, Hct was also lower than preoperatively in all groups (Tukey HSD test, *p* < 0.05). On the first postoperative day, Hct decreased significantly in Group C (10%) compared with Group A (7.4%) or Group B (7.5%) (Tukey HSD test, *p* < 0.001). On the third postoperative day, there was no difference between Groups C (7%), A (7.5%) and B (7.2%).

Fourteen transfusions were required in the control Group C of 85 patients, while only 5 of the 65 patients in Group A who received TXA only and only 7 of the 59 patients of Group B that received TXA plus vancomycin required transfusions. These transfusion rates did not differ significantly between groups (chi-square test, *p* = 0.319). One deep infection and one DVT were observed in control Group C, while no infection or DVT occurred in Group A, while in Group B, there was one deep infection, managed with a two-stage revision protocol. The numbers of adverse events were too low to make comparisons (Table 3).

### 3.2. Total Knee Arthroplasty Results

**Figure 2:**
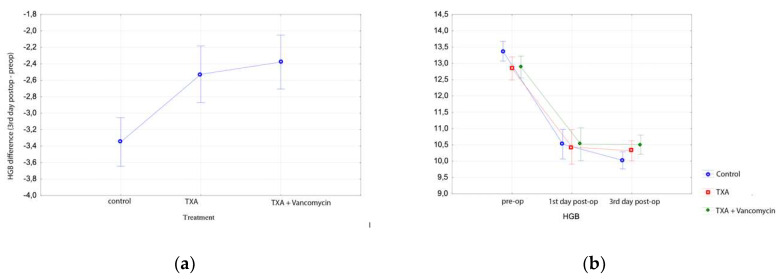
Results in total knee arthroplasty patients: (**a**) Hemoglobin difference between preoperative and 3rd day postoperative value in the control and treatment groups of total knee arthroplasty; (**b**) hemoglobin levels before total knee arthroplasty, at first and third postoperative days in the control and treatment groups.

Hgb dropped significantly on the 3rd postoperative day in all three groups (Tukey HSD test, *p* < 0.001 for all groups). Hgb drop was greater in Group C than in Groups A and B. (Table 2, Figure 2). Hgb decreased by 2.8 ± 1.1 gr/dL in Group C, while it decreased only 1.9 ± 1.1 in Group A and 1.8 ± 0.9 in Group B, respectively (Tukey HSD, *p* < 0.001 for both comparisons). There was no difference in Hgb drop on the 3rd postoperative day between Groups A and B (Tukey HSD test, *p* = 0.720), supporting again our hypothesis that the addition of vancomycin does not affect the topical action of TXA (Figure 2).

Hct also decreased in all groups postoperatively (Tukey HSD test, *p* < 0.001). In the third postoperative day, Hct decreased significantly in Group C (8.6% ± 3.1%) compared with (5.8% ± 2.6%) in Group A or 5.3% ± 2.5% in Group B (Tukey HSD test, *p* < 0.001 for both comparisons). This significant difference was also observed in the first postoperative day (Tukey HSD test, *p* < 0.001 for both comparisons).

Nine transfusions were required in control Group C of 93 patients, while only one of the 83 patients who received TXA and only one of the 83 patients who received TXA plus vancomycin required transfusions. These transfusions were significantly different between Group C (Fisher’s exact test, *p* = 0.02) and Groups A or B. Again, it appears that the topical application of vancomycin did not affect the anti-thrombolytic activity of TXA.

One deep infection, one superficial and two complex regional pain syndromes, in which infection was ruled out, were observed in control Group C. One deep infection and four superficial wound healing delays occurred in Group A. In Group B, one acute deep infection and one superficial wound healing delay were identified. One of the deep infections was caused by *Staph. aureus* not resistant to vancomycin. Deep infections were managed with a two-stage revision protocol. Wound-healing problems were managed with IV antibiotics for three days or surgical debridement ± negative pressure therapy. The acute deep infection was managed with debridement and IV antibiotics and implant retention after polyethylene exchange. The number of adverse events in each group was too low to make comparisons (Table 3).

## 4. Discussion

This study demonstrates that the application of tranexamic acid inside the joint space after hip and knee replacement surgery is safe and effective even after vancomycin powder is added. Intra-articular use of TXA is considered to be effective and comparable to IV TXA administration [23]. A meta-analysis by Zhang et al. has shown no difference between the two ways of TXA application in THAs [4]. Network meta-analysis revealed that the best strategy is to combine IV pre-incision TXA with intra-articular application [24,25,26]. However, this would not be applicable to patients with increased risk for thromboembolic events, who are usually excluded from randomised trials.

All THAs were carried out through the posterior approach commonly preferred because it does not result in limping and the exposure of both the acetabulum and the femur is safe and adequate, although the risk of dislocation is slightly increased [27]. The findings of this study demonstrated that combining the TXA and vancomycin powder into the joint space before wound closure reduced the drop of Hgb in total hip and total knee arthroplasty. We can postulate that the end result of the topical action of TXA was the reduction of local bleeding and the preservation of Hgb levels and this is achieved despite the concomitant presence of the vancomycin power into the joint space. This reduction was translated into reduced blood loss and transfusion needs only for TKAs. Other studies have produced conflicting results, especially for the TKAs [22,23,28,29]. The network meta-analysis of Fillingham et al. [26] revealed that the topical application of TXA significantly reduced the transfusions in TKAs. A recent meta-analysis of series with THAs showed that only combined IV and intra-articular administration significantly reduced the need for blood transfusions [24].

For patients with increased thromboembolic risk, the IV use of TXA is not recommended and it is safer when TXA is applied into the joint [8]. Moskal et al. found an increased risk for DVT compared to placebo when IV TXA was used in THAs. However, Yang did not reach such a conclusion in their meta-analysis of combined topical and IV versus intra-articular or IV administration [2,3]. Plasma concentration after local use of TXA into the joint was found 70% lower compared to IV application [28]. As a result, not only is the risk for thromboembolic events minimised but also the risk for seizures is reduced, especially when high doses are administrated [6,7]. Finally, TXA can exert the anti-inflammatory properties on the topical tissues when used into the wound and may have a positive effect on the rehabilitation and in reducing pain [9].

The use of vancomycin powder into the joint has been used not only in spine surgery but also in foot and ankle surgery and trauma [30,31] with encouraging results. The advantage of topical vancomycin is the possibility to reach higher concentrations, calculated 100 to 1000 higher than minimum inhibitory concentration (MIC) (2 μgr/mL) for the first four days [13]. There is skepticism that vancomycin may result in the development of resistance, but this has not occurred up to date in a study with 2800 patients. In that study, none of the *Staph. aureus* isolates from patients that received topical vancomycin had MIC > 2 μgr/mL [13]. In addition, basic research has proven that the wear rate of the polyethylene is not influenced by the vancomycin [32]. One disadvantage after the use of topical vancomycin might be the development of seromas.

A recent review of low-quality studies suggested that the intra-wound vancomycin may reduce the risk of periprosthetic joint infection in primary total joint arthroplasty [33]. Other studies did not generate similar results [34,35]. Given the cost-effectiveness of the topical use of vancomycin, a randomised controlled trial is needed [36], although it would be difficult to implement because over 2000 patients per group would be required to reveal a 50% decrease in infection rate. The number of infections in all groups was small and obviously underpowered to make useful comparisons. Twenty-three patients were lost to follow-up. It would be unlikely that these patients would affect the results of the study because the number is small, and the same percentage of patients in each group were lost to follow-up. Lastly, we believe that any infections in patients lost to follow-up would probably be managed in our department because it is the only referral center for periprosthetic joint infections in the central part of the country.

Limitations of the study include the lack of a separate vancomycin-only group, but this was considered unethical given the proven efficacy of tranexamic acid to reduce blood loss. Another limitation is that the study was underpowered to reveal any difference in the infection rate between groups proving the safety of topical co-administration of vancomycin with TXA. Still, an ongoing laboratory study is addressing this problem and exploring the inhibition of *Staph. aureus* growth by the vancomycin plus TXA mix. Even if a reduction in superficial or deep infections is not proven, the surgeons may safely use this combination to mitigate the risk of this devastating adverse event of periprosthetic joint infection. In conclusion, the use of topical administration of vancomycin powder into the joint space does not reduce the effect of tranexamic acid in reducing the blood loss in total knee and hip arthroplasties.

## Figures and Tables

**Table 1:** Demographics of the patients.

	Number of Patients	Gender (Females/Males) (*n*)	Age (Mean ± SD)
Total knee replacements			
TXA (Group A)	83	69/14	71.3 ± 5.7
TXA + vancomycin (Group B)	83	66/17	70.3 ± 7.6
Control (Group C)	93	76/17	70.5 ± 5.5
		Chi square test, *p* = 0.565	ANOVA test, *p* = 0.556
Total hip replacements			
TXA (Group A)	65	42/23	64 ± 12.5
TXA + vancomycin (Group B)	59	35/24	62.1 ± 11.9
Control (Group C)	85	52/33	62.5 ± 11.3
		Chi square test, *p* = 0.332	ANOVA test, *p* = 0.631

**Table 2:** Hemoglobin and hematocrit values of control group (C) and treatment groups (tranexamic and tranexamic plus vancomycin) (Groups A and B) before total hip arthroplasty and during first and third post-operative day.

	Hgb Preop (gr/dL) (Mean, SD)	Hgb 1st Day Post-op (gr/dL) (Mean, SD)	Hgb 3rd Day Post-op (gr/dL) (Mean, SD)	Hct Preop (%) (Mean, SD)	Hct 1st Day Post-op (%) (Mean, SD)	Hct 3rd Day Post-op (%) (Mean, SD)
THA						
Group A	12.8 ± 1.0	10.4 ± 1.3	10.3 ± 1.2	38.6 ± 2.8	31.2 ± 3.9	31.0 ± 3.7
Group B	12.9 ± 1.2	10.5 ± 1.5	10.5 ± 1.3	38.9 ± 3.5	30.9 ± 4.0	32.2 ± 2.3
Group C	13.3 ± 1.6	10.5 ± 2.7	10.0 ± 1.1	40.5 ± 3.5	30.3 ± 4.0	33.8 ± 3.6
TKA						
Group A	13.4 ± 1.0	11.7 ± 1.2	11.5 ± 1.4	40.3 ± 2.8	35.0 ± 3.1	34.5 ± 3.6
Group B	13.2 ± 1.0	11.3 ± 1.3	11.4 ± 1.2	39.5 ± 2.8	34.0 ± 3.5	34.2 ± 3.5
Group C	13.1 ± 1.4	10.8 ± 1.5	10.3 ± 1.2	39.4 ± 3.7	32.4 ± 4.2	30.8 ± 3.4

**Table 3:** Transfusions and adverse events in total hip arthroplasties (THAs) and total knee arthroplasties (TKAs).

	Transfusions	Delayed Wound Healing	Superficial Infections	Deep Infections	DVTs
THA					
Group A (*n* = 65)	5	0	0	0	0
Group B (*n* = 59)	7	0	0	1	0
Group C (*n* = 85)	14	0	0	1	1
TKA					
Group A (*n* = 83)	1	4	1	1	0
Group B (*n* = 83)	1	1	0	1	0
Group C (*n* = 93)	9	1	0	1	0
